# Predictors of survival outcomes among patients with gastric cancer in a leading tertiary, teaching and referral hospital in Kenya

**DOI:** 10.1002/cam4.5275

**Published:** 2022-09-29

**Authors:** Amsalu Degu, Peter N. Karimi, Sylvia A. Opanga, David G. Nyamu

**Affiliations:** ^1^ Department of Pharmaceutics and Pharmacy Practice, School of Pharmacy and Health Sciences United States International University–Africa Nairobi Kenya; ^2^ Department of Pharmacy, Faculty of Health Sciences University of Nairobi Nairobi Kenya

**Keywords:** gastric cancer, mortality, predictors, survival outcomes

## Abstract

**Introduction:**

The incidence of gastrointestinal malignancies in Kenya is increasing, although there is a paucity of data on survival outcomes among gastric cancer patients. Hence, this study aimed to assess survival outcomes among adult gastric cancer patients at Kenyatta National Hospital.

**Methods:**

A retrospective cohort study design was used to assess the survival outcomes among 247 gastric cancer patients. All medical records of adult (≥18 years) gastric cancer patients with complete medical records of diagnosis, stage of cancer, and treatment regimen in the study setting in the last 5 years (2016–2020) were included. A simple random sampling technique was employed to select the study participants. Data were collected using a data abstraction tool composed of socio‐demographic and clinical characteristics. Survival outcomes were reported as the percentage of mortality, mean survival estimate, and mean cancer‐specific survival. The data were entered and analyzed using version 20.0 SPSS statistical software. The mean survival estimates and predictors of mortality were computed using the Kaplan–Meier and Cox regression analysis.

**Results:**

The study showed that 33.3% (64) had new distant metastasis, and 42.1% (104) had disease progression. Besides, the mortality rate was high (33.6%), and 14.6% and 7.7% of patients had complete and partial responses, respectively. The five‐year survival was 32.7% among gastric cancer patients. Comorbidity (*p* = 0.014), advanced‐stage diseases (*p* = 0.03), chemotherapy (*p* = 0.008), and gastrectomy (*p* = 0.016) were significant determinants of mortality.

**Conclusions:**

A significant proportion of patients had distant metastasis, disease progression, and a low five‐year survival rate. Hence, early cancer‐screening programs are indispensable to circumvent disease progression and improve survival outcomes.

## 1 INTRODUCTION

Globally, the cancer burden is estimated to be 18.1 million new cases and 9.6 million deaths in 2018.^1^ Approximately 70% of cancer deaths occur in developing countries.^2^ By 2030, there will be 23.6 million new cancer cases worldwide per year.^3^ This growing trend of cancer incidence has spurred more work on cancer prevention and treatments.^4^

Colorectal, gastric, esophageal, and liver cancers are the most frequently diagnosed gastrointestinal cancers globally.^5^ Moreover, gastrointestinal cancers account for 26% of the global cancer incidence and 35% of all cancer deaths.^6^

Although the incidence of some gastrointestinal cancer types has diminished, they continue to pose substantial challenges to public health.^6^ Gastric cancer incidence has decreased dramatically in the past 50 years because of a decrease in *Helicobacter pylori* infection, smoking habits, and the use of processed meat.^7^ Complete eradication of the organism after treatment reduced the risk.^8^ Although the treatment outcomes of advanced gastric cancer have improved with the advancement of chemotherapy and surgery, there are still areas for improvement in achieving substantial beneficial effects to increase patient survival.^9^ Gastric cancer is an age‐related disease and is commonly diagnosed in the older population. Therefore, achieving the desired outcomes of treatment is difficult due to age‐related reduced organ function.^10^

In contrast, in developed countries, despite the incidence of gastric cancers steadily falling in the past 50 years, treatment outcomes remain poor, mainly due to the diagnosis of the disease at an advanced stage. Besides, the prognosis for advanced stages of gastrointestinal cancer remains very poor, despite extensive attempts to improve treatment, including the development of new medicines. Therefore, there is an immediate need for effective disease management to ensure that patients are given the most suitable care.^11^ The treatment outcome of cancer patients is poor in sub‐Saharan Africa compared to other regions due to the lack of adequate treatment and diagnostic facilities.^12^

There are many inconclusive pieces of evidence of survival outcomes of gastric cancer patients globally. Besides, the studies available in East Africa are few and non‐comprehensively covered. Thus, this study aimed to assess survival outcomes among gastric cancer patients at Kenyatta National Hospital (KNH).

## 2 METHODS

### 2.1 Study design, setting and period

A single‐arm retrospective cohort study was employed to assess the survival outcomes among adult gastric cancer patients. The study was conducted at the Oncology Department of KNH. This health facility is the biggest teaching and referral hospital in Kenya and East Africa, with a 2000 bed capacity. The hospital is at the top of Kenya's referral system for the health sector and has adequate infrastructure for managing patients with different types of cancers. All included medical records of gastric patients were retrospectively reviewed from the time of diagnosis to the last follow‐up visit to the hospital. This review of medical records was conducted from 27th September 2021 to 31st January 2022.

### 2.2 Target population

All adult patients (≥18 years) with a histologically confirmed diagnosis of gastric cancers treated in the last 5 years (2016–2020) at the Oncology Department of KNH were the target population for this study design.

### 2.3 Eligibility criteria

#### 2.3.1 Inclusion criteria

- All adult patients (≥18 years) with a histologically confirmed diagnosis of gastric cancer in the study setting in the last 5 years (2016–2020).
- All adult patients (≥18 years) with complete medical records of diagnosis, stage of cancer, and treatment regimen in the study setting in the last 5 years (2016–2020).

#### 2.3.2 Exclusion criteria

- All medical records of adult gastric patients (≥18 years) with incomplete medical records of diagnosis, stage of cancer, and treatment regimens in the last 5 years (2016–2020).

### 2.4 Sample size determination

Yamane's formula was employed to determine the sample size.^13^

$$n=\frac{N}{1+N*\left(e^{2}\right)}$$

Where n = the estimated sample size, N = Population, e = the level of significance at 95% confidence level (0.05). According to the Health Information Department of KNH, around 508 gastric cancer patients were treated in the study setting in the last 5 years (2016–2020). Accordingly, the estimated sample size with a 10% contingency for the patient's insufficient medical records was provided with a final sample size of 247 gastric cancer patients.

### 2.5 Sampling techniques

A simple random sampling technique using the lottery method was employed to select the medical records of the patients.^14^

### 2.6 Data collection

The data collectors randomly selected eligible medical records from the list of gastric cancer patient files. The structured data abstraction tool was designed as per previous studies with some modifications.^15^, ^16^, ^17^, ^18^, ^19^, ^20^ Then, data on sociodemographic, clinical characteristics, and histological types of cancer were collected. Besides, cancer‐specific survival (time to death or last follow‐up period) was calculated by subtracting the primary gastric cancer diagnosis date from the time of the last follow‐up period or death. The overall mortality rate was also determined by dividing the total number of patients who died in the follow‐up period by the total sample size included in the study. The five‐year survival rate was computed as per the previously published methods described by Nohrman.^21^ The response status after treatment (complete, partial, progression, no response) was determined using the documented interval scan of the tumor in reference to the Response Evaluation Criteria in Solid Tumors (RECIST) revised guideline with slight modifications.^22^

### 2.7 Pretest study

Before initiating the actual study, a pretest was done in 5% of the sample size to ensure the data collection instruments' validity. After pretesting, all appropriate changes were made to the data collection instruments before executing the actual study.

### 2.8 Data analysis

The data were entered, cleaned, and analyzed using SPSS version 20.0 statistical software. Percent, frequency, mean, and standard deviation were used to summarize the study variables. The survival outcome was estimated using the Kaplan–Meier analysis. Bivariate and multivariate Cox regression analysis was used to investigate the potential predictors of survival outcomes. A *p*‐value of ≤0.05 was considered statistically significant.

### 2.9 Definition of terms

Survival outcome was reported in terms of mortality, cancer‐specific survival, complete response and partial response, progression of the diseases and nonresponse. Cancer‐specific survival is the time from the date of primary cancer diagnosis to the date of cancer‐related death or last follow‐up. Complete response, partial response, progressive disease and nonresponse is defined as no evidence of disease on repeat scanning, reduction in tumor size of ≥30%, failure to achieve remission and increased tumor size (≥20% increase or the appearance of one or more new lesions during the interval computerized tomography [CT] scan) despite therapy, respectively.

## 3 RESULTS

### 3.1 Sociodemographic characteristics of the study participants

A total of 247 eligible gastric cancer patients were involved in the study, and their median age was 60.0 years (interquartile range 51–69 years). Most (161, 65.2%) of the study participants were males. The median follow‐up time was 5 months (range: 1–62 months) (Table 1).

**TABLE 1:** Socio‐demographic characteristics of the study participants

Variable	Frequency (%)
Age (in years)	
< 60 years	122 (49.4)
≥60 years	125 (50.6)
Gender	
Male	161(65.2)
Female	86 (34.8)
Marital status	
Single	55 (22.3)
Married	187 (75.7)
Widowed	5 (2)
Educational status	
Primary	172 (69.6)
Secondary	39 (15.8)
Tertiary	15 (6.1)
Nonformal	21 (8.5)
Occupational status	
Housewife	20 (8.1)
Government employee	34 (13.8)
Unemployed/Retired	42 (17)
Self‐employed	75 (30.4)
Other	76 (30.8)
Family history of cancer	
No	241 (97.6)
Yes	6 (2.4)

*Note*: Other: Student, Contractor, Driver, Teacher, Artisan, house help.

### 3.2 Clinical characteristics of the study participants

Histologically, most patients (244, 98.8%) had adenocarcinoma and stage II and III TNM staging at diagnosis. In the study setting, 22.3% (55) of gastric cancer patients had evidence of distant metastasis at the time of diagnosis, where the liver was the most (34, 13.8%) common site of metastasis. Approximately three‐fifths (147, 59.5%) of the patients had co‐current co‐morbidities, while the majority (97, 39.3%) had only one co‐existing condition. Anemia (58, 23.5%), hypertension (31, 12.6%), and peptic ulcer disease (25, 10.1%) were the leading co‐morbidities among gastric cancer patients (Table 2).

**TABLE 2:** Clinical characteristics of the study participants

Variable	Frequency	Percent
Histological type of cancer		
Adenocarcinoma	244	98.8
Squamous cell carcinoma	3	1.2
Stage of cancer		
Stage I	6	2.4
Stage II	93	37.7
Stage III	93	37.7
Stage IV	55	22.3
Co‐morbidity		
Present	147	59.5
Absent	100	40.5
Number of co‐morbidities		
One	97	39.3
Two	30	12.1
≥Three	20	8.1
Type of co‐morbidity		
Anemia	58	23.5
Hypertension	31	12.6
Peptic ulcer disease	25	10.1
Ascites	17	6.9
Acute kidney injury	16	6.5
Diabetes mellitus	15	6.1
Upper gastrointestinal bleeding	10	4.0
Gastric outlet obstruction	10	4.0
Chronic kidney disease	6	2.4
Deep vein thrombosis	6	2.4
Obstructive jaundice	4	1.6
Benign prostatic hyperplasia	4	1.6
Heart failure	4	1.6
Asthma	3	1.2
Hypovolemic shock	2	0.8
Pulmonary embolism	3	1.2
Pneumonia	2	0.8
Retroviral disease	2	0.8
Adenoid cystic carcinoma	2	0.8
Alcoholic liver disease	1	0.4
Hydronephrosis	1	0.4
Pancytopenia	1	0.4
Acute pancreatitis	1	0.4
Chronic pancreatitis	1	0.4
Gastroesophageal reflux disease	1	0.4
Distance metastasis at diagnosis	55	22.3
Liver	34	13.8
Lung	8	3.2
Liver & pancreas	3	1.2
Liver & lung	3	1.2
Liver & brain	2	0.8
Liver & spleen	1	0.4
Liver, pancreas & Adrenal gland	1	0.4
Liver & bone	1	0.4
Peritoneal cavity	1	0.4
Bone	1	0.4

In terms of management, chemotherapy was the most (150, 60.7%) frequently used treatment modality among gastric cancer patients, where the combination of folinic acid, fluorouracil, and oxaliplatin chemotherapy regimen accounted for the predominant proportion (39, 15.8%). Gastrectomy and radiotherapy accounted for 46.2% (114) and 10.9% of the enrolled patients, respectively. However, in 15% of gastric patients, the symptomatic management approach was employed in the study setting.

### 3.3 Survival outcomes of the study participants

Among the 247 patients, 33.3% (64) had evidence of new distant metastasis in the follow‐up period. Of those cases, metastasis to the liver and lung were the most common sites of spread. Nonetheless, metastasis to the ovary, bone, and multiorgan metastases were the least common sites of spread during the last follow‐up period.

**FIGURE 1:**
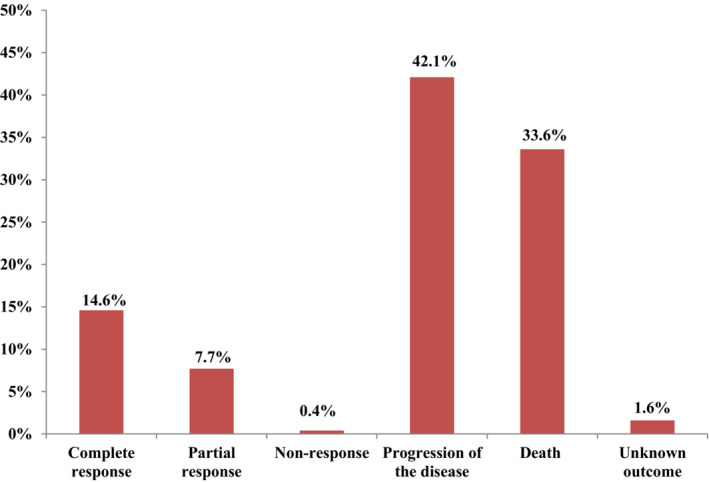
The response status of patients during the last follow‐up period.

The present study showed that 42.1% (104) of the patients had disease progression during the follow‐up period. Besides, the mortality rate was 33.6% (83), even though 1.6% had unknown outcomes. Furthermore, 14.6% and 7.7% of patients had complete responses and partial responses, respectively (Figure 1). The five‐year survival of gastric cancer patients was 32.7%, with a mean cancer‐specific survival of 27.6 ± 3.1 months.

**FIGURE 2:**
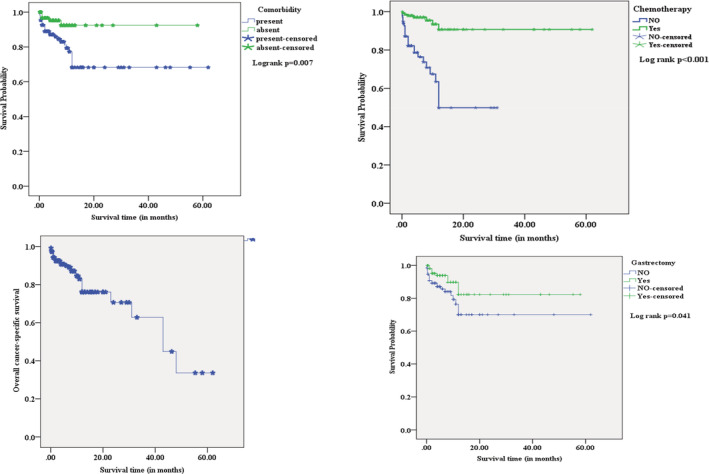
The overall cancer‐specific survival of gastric cancer patients with co‐morbidities and treatment modalities.

The study showed no statistically significant mean differences in survival estimates between age groups, gender, and histological type of cancer compared to their respective counterparts. However, the mean survival estimates of gastric cancer patients with co‐morbidity (44.4 months), advanced stage of disease (40.7 months), and distant metastasis (13.3 months) were significantly shorter than their counterparts. In contrast, patients treated with surgery (49.1 months) and chemotherapy (56.9 months) had a longer duration of mean survival estimates than their counterparts. On the other hand, radiotherapy‐treated patients did not significantly differ in mean survival estimates compared to patients without radiotherapy. Comparatively, chemotherapy‐treated gastric cancer patients had the longest duration of mean survival estimates than other treatment modalities (Table 3 and Figure 2).

**TABLE 3:** Mean survival time estimates among the study participants

Variables	Mean survival estimate (months) ± standard error (95% CI)	Log‐rank test (*p*‐value)
Age (years)		0.532
< 60 years	46.4 ± 3.5 (39.5–53.2)	
≥ 60 years	46.1 ± 2.6 (40.9–51.3)	
Gender		0.918
Male	46.4 ± 3.4 (39.7–53.2)	
Female	47.9 ± 2.9 (42.2–53.6)	
Co‐morbidity		0.007*
Present	44.4 ± 3.1 (38.3–50.6)	
Absent	53.9 ± 1.9 (50.2–57.6)	
Stage of cancer		0.031*
Early‐stage (I &II)	53.1 ± 3.2 (46.7–59.4)	
Advanced stage (III & IV)	40.7 ± 2.9 (34.9–46.5)	
Histological type of cancer		0.838
Adenocarcinoma	49.1 ± 2.4 (44.4–53.7)	
Squamous cell carcinoma	17.3 ± 2.2 (13.1–21.6)	
Distant metastasis at diagnosis		< 0.001*
Yes	13.3 ± 1.6 (10.1–16.4)	
No	54.6 ± 2.2 (50.4–58.8)	
Distant metastasis in the follow‐up period		0.118
Yes	43.9 ± 2.7 (38.6–49.2)	
No	53.5 ± 3.7 (46.3–60.8)	
Treatment regimen		
Chemotherapy		< 0.001*
No	18.7 ± 2.0 (14.7–22.6)	
Yes	56.9 ± 2.0 (52.9–62.9)	
Gastrectomy		0.041*
No	45.3 ± 3.5 (38.5–52.1)	
Yes	49.1 ± 2.9 (43.4–54.9)	
Radiotherapy		0.500
No	45.2 ± 2.5 (40.2–49.9)	
Yes	52.1 ± 5.3 (41.7–62.5)	
Symptomatic therapy		< 0.001*
No	55.1 ± 2.1 (51.0–59.1)	
Yes	6.7 ± 0.9 (4.9–8.5)	

* Statistically significant *p*‐value ≤0.05.

Patients with co‐existing co‐morbidities had a 3.3 times more risk of dying than those without co‐morbidities (AHR: 3.3, 95% CI: 1.3–8.7, *p* = 0.014). Patients with advanced‐stage diseases and distant metastasis at diagnosis had 2.4 (AHR: 2.4, 95% CI: 1.1–5.2, *p* = 0.03) and 7.7 (AHR: 7.7, 95% CI: 3.1–19.1, *p* < 0.001) times more risk of dying than their counterparts, respectively. Gastric cancer patients who did not receive chemotherapy (AHR: 5.2, 95% CI: 1.5–17.8, *p* = 0.008) and gastrectomy (AHR: 1.7, 95% CI: 1.1–2.6, *p* = 0.016) had a statistically significant higher risk of dying compared to patients who received the respective treatment modalities. In contrast, radiotherapy did not show a statistically significant difference in mortality risk among gastric cancer patients. Nonetheless, patients treated with symptomatic management had a higher risk of dying than patients without symptomatic management. Age, gender, histological type of cancer, and distant metastasis in the follow‐up period were not statistically significant determinants of survival outcomes among gastric cancer patients (Table 4).

**TABLE 4:** Determinants of mortality among the study participants

Variable	Bivariate analysis		Multivariate analysis	
	CHR (95% CI)	*p*‐value	AHR (95% CI)	*p*‐value
Age (years)				
< 60 years	1		1	
≥ 60 years	0.8 (0.4–1.6)	0.537	0.9 (0.4–1.9)	0.741
Gender				
Male	1		1	
Female	0.9 (0.5–1.9)	0.919	2.1 (0.9–4.8)	0.067
Co‐morbidity				
Absent	1		1	
Present	3.4 (1.3–8.8)	0.012*	3.3 (1.3–8.7)	0.014*
Types of co‐morbidity				
Hydronephrosis				
No	1		1	
Yes	12.6 (1.6–3.6)	0.014*	31.4 (3.9–10.2)	0.001*
Hypovolemic shock				
No	1		1	
Yes	8.3 (1.1–3.3)	0.039*	20.7 (2.6–12.5)	0.004*
Anemia				
No	1		1	
Yes	2.9 (1.5–5.8)	0.002*	3.8 (1.8–7.8)	<0.001*
Acute kidney injury				
No	1		1	
Yes	2.5 (1.0–6.1)	0.043*	4.2 (1.6–10.7)	0.003*
Ascites				
No	1		1	
Yes	3.7 (1.5–9.1)	0.005*	5.1 (1.9–13.1)	0.001*
Stage of cancer				
Early‐stage (I &II)	1		1	
Advanced stage (III & IV)	2.3 (1.1–5.2)	0.038*	2.4 (1.1–5.2)	0.03*
Histological type of cancer				
Adenocarcinoma	1		1	
Squamous cell carcinoma	1.2 (0.2–9.1)	0.840	0.2 (0.1–1.5)	0.103
Distant metastasis at diagnosis				
No	1		1	
Yes	6.4 (3.2–12.8)	<0.001*	7.7 (3.1–19.1)	<0.001*
Distant metastasis in the follow‐up period				
No	1		1	
Yes	0.5 (0.2–1.2)	0.130	0.6 (0.2–1.8)	0.326
Treatment regimen				
Chemotherapy				
Yes	1		1	
No	7.5 (3.2–17.3)	<0.001*	5.2 (1.5–17.8)	0.008*
Gastrectomy				
Yes	1		1	
No	2.1 (1.1–4.5)	0.04*	1.7 (1.1–2.6)	0.016*
Radiotherapy				
Yes	1		1	
No	1.5 (0.5–4.9)	0.507	0.8 (0.2–3.2)	0.754
Symptomatic therapy				
Yes	1		1	
No	0.08 (0.1–0.2)	<0.001*	0.3 (0.1–0.9)	0.034*

Abbreviations: AHR, Adjusted hazard ratio; CHR, Crude hazard ratio.

* Statistically significant *p*‐value ≤0.05.

In the subgroup analyses, in males, adults (<60 years), and older patients (>60 years), chemotherapy and gastrectomy treatment modalities were statistically significant predictors of mortality. However, chemotherapy (*p* = 0.001) was the only significant predictor of mortality in female gastric cancer patients (Table 5). In the advanced stage of gastric cancer (stage III &IV), chemotherapy (*p* ≤ 0.002), gastrectomy (*p* = 0.002), radiotherapy (*p* = 0.01) and co‐morbidity (*p* = 0.04) were the significant predictors of mortality. Nonetheless, gastrectomy (*p* = 0.02) treatment was the only significant predictor in the early stage (I &II) gastric cancer patients (Table 6).

**TABLE 5:** Subgroup analysis of the determinants of mortality based on age and gender among the study participants

Variables	Categories	Bivariate analysis		Multivariate analysis	
		CHR (95% CI)	*p*‐value	AHR (95% CI)	*p*‐value
< 60 years	Comorbidity				
	Absent	1		1	
	Present	3.5 (1–11.9)	0.051	1.3 (0.3–5.2)	0.678
	Early‐stage (I &II)	1		1	
	Advanced stage (III & IV)	0.3 (0.1–1.0)	0.06	0.7 (0.2–2.2)	0.519
	Chemotherapy				
	Yes	1		1	
	No	4.5 (1.5–15.9)	0.007*	11.7 (3.1–12.0)	<0.001*
	Gastrectomy				
	Yes	1		1	
	No	2.5 (0.9–6.5)	0.066	3.7 (1.3–10.4)	0.012*
	Radiotherapy				
	Yes	1		1	
	No	0.9 (0.2–2.5)	0.386	1.1 (0.2–3.3)	0.744
≥ 60 years	Co‐morbidity				
	Absent	1		1	
	Present	3.2 (0.7–14.5)	0.123	1.5 (0.3–7.5)	0.624
	Early‐stage (I &II)	1		1	
	Advanced stage (III & IV)	0.6 (0.2–1.8)	0.371	0.5 (0.1–1.5)	0.200
	Chemotherapy				
	Yes	1		1	
	No	10.5 (3.0–20.2)	<0.001*	10.1 (2.6–20.2)	0.001*
	Gastrectomy				
	Yes	1		1	
	No	1.8 (0.6–5.9)	0.305	4.1(1.2–14.2)	0.028*
	Radiotherapy				
	Yes	1		1	
	No	0.8 (0.2–2.7)	0.664	1.2 (0.4–4.5)	0.788
Male	Co‐morbidity				
	Absent	1		1	
	Present	2.1 (0.7–6.3)	0.178	0.6 (0.2–2.2)	0.446
	Early‐stage (I &II)	1		1	
	Advanced stage (III & IV)	0.4 (0.1–1.1)		0.4 (0.2–1.2)	0.113
	Chemotherapy				
	Yes	1		1	
	No	7.2 (2.4–21.5)	<0.001*	13.6 (3.8–30.2)	<0.001*
	Gastrectomy				
	Yes	1		1	
	No	2.7 (1.2–7.5)	0.05*	5.4 (1.9‐15.5)	0.001*
	Radiotherapy				
	Yes	1		1	
	No	1.3 (0.6–2.8)	0.441	1.5 (0.7–3.2)	
Female	Comorbidity				
	Absent	1		1	
	Present	8.3 (1.1–10.2)	0.042*	2.3 (0.6‐3.4)	0.127
	Early‐stage (I &II)	1		1	
	Advanced stage (III & IV)	0.5 (0.1–1.9)		1.0 (0.2–4.5)	0.963
	Chemotherapy				
	Yes	1		1	
	No	8.4 (2.3–23.3)	0.001*	10.3 (2.5–10.0)	0.001*
	Gastrectomy				
	Yes	1		1	
	No	1.4 (0.4–4.4)	0.569	2.9 (0.8–10.3)	0.105
	Radiotherapy				
	Yes	1		1	
	No	1.0 (0.4–2.9)	0.939	1.3 (0.4–4.5)	0.665

Abbreviations: AHR, Adjusted hazard ratio; CHR, Crude hazard ratio.

* Statistically significant *p*‐value ≤0.05.

**TABLE 6:** Subgroup analysis of the determinants of mortality based on the stages of the disease among gastric cancer patients

Variables	Categories	Bivariate analysis		Multivariate analysis	
		CHR (95% CI)	*p*‐value	AHR (95% CI)	*p*‐value
Early stage (I&II)					
	Chemotherapy				
	Yes	1		1	
	No	2.5 (0.6–10.3)	0.200	3.4 (0.7–15.4)	0.114
	Gastrectomy				
	Yes	1		1	
	No	1.9 (0.5–8.3)	0.352	2.5 (0.5–13.2)	0.02*
	Radiotherapy				
	Yes	1		1	
	No	0.4 (0.1–2.1)	0.296	0.6 (0.1–3.4)	0.535
	Co‐morbidity				
	Absent	1		1	
	Present	1.1 (0.3–4.6)	0.912	0.7 (0.1–3.3)	0.640
Advanced stage (III&IV)					
	Chemotherapy				
	Yes	1		1	
	No	13.8 (4.1–20.2)	<0.001*	16.6 (4.5–20.2)	<0.002*
	Gastrectomy				
	Yes	1		1	
	No	1.9 (0.8–4.6)	0.140	4.2 (1.7–10.4)	0.002*
	Radiotherapy				
	Yes	1		1	
	No	3.5 (0.5–26.6)	0.002*	3.2 (0.4–24.2)	0.01*
	Co‐morbidity				
	Absent	1		1	
	Present	6.2 (1.5–20.2)	0.013*	2.7 (1.3–7.9)	0.04*

Abbreviations: AHR, Adjusted hazard ratio; CHR, Crude hazard ratio.

* Statistically significant *p*‐value ≤0.05.

## 4 DISCUSSION

Although early detection of gastric cancer can lead to long‐term survival,^15^ there is much inconclusive evidence about the survival outcome of gastrointestinal cancer patients globally. In addition, some countries have an increased incidence of gastric cancer in the younger population.^23^ However, the studies available in the East African setting are minimal in assessing survival outcomes after treatment in gastric cancer patients. Therefore, this study highlights the determinants of survival outcomes among gastric cancer patients.

At the time of diagnosis, 22.3% of patients with gastric cancer exhibited distant metastasis, with the liver and lung being the most prevalent sites of metastasis. Besides, 33.3% of patients had evidence of new distant metastasis in the follow‐up period, which can worsen survival. This could probably be linked to late diagnosis and delayed initiation of optimal treatment in our setting.

The mortality rate was significantly higher than Africa's estimated mortality rate (3.8%).^24^ This high mortality rate could be due to the late diagnosis and lack of adequate facilities for treatment in the study setting. Despite this, 14.6% and 7.7% of patients had complete and partial responses, respectively, contrasting findings in the Cameroon study, which demonstrated a 70.8% mortality.^16^ Therefore, educational programs should be implemented nationally about the essence of early diagnosis and treatment of gastric cancer patients for improved survival outcomes.

Previous studies showed a mean five‐year survival rate of 15.5% among gastric cancer patients with a reduction in the survival trends from the first to the fifth year.^15^ In addition, a Cameronian study also reveals a 4.6% five‐year survival rate.^16^ A previous systematic review shows a relatively poor five‐year survival rate in Africa compared to Korean and Japanese gastric cancer patients.^25^ In contrast, the findings in our study reported a significantly higher five‐year survival rate (32.7%) despite the survival rate being reduced from 1 year to the fifth year after the diagnosis of the disease. However, awareness creation education programs about early signs and symptoms of gastric cancer, optimal management and availability of anticancer drugs in all cancer treatment centers are essential to enhance overall survival in our setting.

The mean cancer‐specific survival of gastric cancer patients (27.6 months) was slightly higher than the Cameronian study (5.91 months).^16^ On the other hand, a Turkish (51 months) and Nigerian (13.6 months) study reports a longer median overall survival.^18^, ^19^ This relatively lower mean survival time in our setting could probably be linked to delay in treatment initiation and late diagnosis as most (60%) of the patients had locally advanced and metastatic disease. Therefore, early gastric cancer screening should be regularly implemented in our setting to improve overall survival in these populations.

The study shows no statistically significant mean differences in survival estimates between age groups, gender, and histological type of cancer compared to their counterparts. Contrastingly, a previous study showed that female and younger patients had shorter and longer survivals, respectively.^26^ However, the mean survival estimates of gastric cancer patients with co‐morbidity (44.4 months), advanced stage of disease (40.7 months), and distant metastasis (13.3 months) were significantly shorter than their counterparts. Therefore, optimal management with frequent follow‐up of these patients is essential to improve survival and health‐related quality of life. Likewise, patients with metastatic gastric cancer had poor survival outcomes despite the efficacy of current chemotherapy in treating gastric cancer.^27^

In contrast, patients treated with gastrectomy (49.1 months) and chemotherapy (56.9 months) had a longer duration of mean survival estimates than their counterparts. This finding is consistent with another study that shows gastric cancer patients treated with surgery and chemotherapy had the longest median overall survival (14.2 months).^20^ These disparities among the studies in survival time could probably be due to the difference in the cancer stage, co‐morbidity, and age of the study participants. On the other hand, radiotherapy‐treated patients did not significantly differ in mean survival estimates from patients without radiotherapy.

In the sub‐group analysis, chemotherapy (*p* ≤ 0.002), gastrectomy (*p* = 0.002), and radiotherapy (*p* = 0.01) treatment approaches were significant determinants of survival in the advanced stage of gastric cancer (stage III &IV) patients. Nonetheless, gastrectomy (*p* = 0.02) was the only significant determinant of survival in the early stage (stage I & II) gastric cancer patients. Therefore, gastrectomy, radiotherapy, and chemotherapy should be the preferred treatment approaches to improve the survival of advanced‐stage gastric cancer patients (stage III &IV) in our setting. In contrast, gastrectomy should be considered in the early stages of the disease (stages I &II).

The mean survival estimate of squamous cell carcinoma (17.3 months) was shorter than adenocarcinoma (49.1 months), suggesting gastric squamous cell carcinoma had poorer survival outcomes than gastric adenocarcinoma. This could probably be linked to the aggressive nature of squamous cell carcinoma and its frequent diagnosis at the advanced stage.^28^, ^29^ Hence, early screening and aggressive treatment modalities are highly recommended to improve survival in these patient populations. This finding agrees with other studies in China.^30^, ^31^, ^32^

Co‐morbidities have a negative prognostic impact on overall survival.^33^ Correspondingly, gastric cancer patients with co‐existing co‐morbidities had 3.3 times more risk of dying than patients without co‐morbidities. Subgroup analysis revealed that surgery (AHR = 0.18, 95% CI:01–0.4, *p* < 0.001) and chemotherapy (AHR = 0.13, 95% CI:0.1–0.3, *p* < 0.001) treatment modalities can enhance survival outcomes among patients with co‐morbidities. Patients with advanced‐stage diseases and distant metastasis at diagnosis had 2.4 and 7.7 times more risk of dying than their counterparts, respectively. Likewise, Talebi et al study also reported a higher hazard of mortality in the metastatic stage of the disease.^34^ Gastric cancer patients who did not receive chemotherapy and gastrectomy treatment had a statistically significant risk of dying compared to patients who received the respective treatment modalities. This could probably be linked to presentation in late stage and only treated with palliative care or supportive treatments. This is consistent with other studies where gastrectomy and chemotherapy were associated with decreased overall mortality in gastric cancer patients.^35^, ^36^, ^37^ Age, gender, and histological type of cancer were not statistically significant determinants of survival outcomes among gastric cancer patients. In contrast, a Korean study showed an increased hazard of mortality among older gastric cancer patients (≥60 years).^38^ Hence, individualized treatment modalities should be considered in co‐morbid and metastatic gastric cancer patients to reduce mortality.

Although molecular biomarkers are important recent advances in the prognostic prediction of treatment response and monitoring of recurrence of gastric cancer,^39^ they are not commonly used in the African setting due to lack of access to these facilities. Therefore, for better prediction of treatment responses in gastric cancer, cancer treatment centers should be equipped with those diagnostic facilities in African settings.

Currently, radiomics coupled with artificial intelligence are also extensively studied for survival outcomes prediction in gastric cancer^40^, ^41^, ^42^ despite our study not addressing these novel technologies. Hence, these risk prediction technologies are highly recommended due to their better capability to handle massive data compared to traditional statistical methods.

### 4.1 Strengths and limitations of the study

The study's main strength was the comprehensive assessment of survival outcomes of gastric cancer patients in a large sample size with long‐term follow‐up. This was the first report assessing the predictors of survival in gastric cancer patients in the study setting. However, being a retrospective study, the validity of the data will be affected by the accuracy of the documentation in the study setting. In addition, as it was only conducted in a single health care facility, it may not be accurately generalized to the general population, so we recommend a large multi‐center study in future. Although *Helicobacter pylori* infection was a key influencing factor in gastric cancer, we did not address this matter due to incomplete information about the status of this infection among the included medical records of the study participants. Furthermore, there were several inconsistencies in the way of surgery among the study participants. Hence, it was difficult to assess the effect of different surgical approaches on the survival outcomes. We recommend further studies to address these gaps.

## 5 CONCLUSIONS

A significant proportion of gastric cancer patients had distant metastasis and disease progression (42.1%) in the last follow‐up period. Co‐morbidity, advanced‐stage diseases, distant metastasis at diagnosis, chemotherapy, and gastrectomy were statistically significant determinants of survival among gastric cancer patients in our setting. Therefore, a multi‐center study is required to determine survival outcomes in gastric cancer patients reliably.

## AUTHOR CONTRIBUTIONS

**Amsalu Degu:** Conceptualization (equal); data curation (equal); formal analysis (equal); funding acquisition (equal); investigation (equal); methodology (equal); project administration (equal); resources (equal); software (equal); supervision (equal); validation (equal); visualization (equal); writing – original draft (equal); writing – review and editing (equal). **Peter N Karimi:** Conceptualization (equal); data curation (equal); formal analysis (equal); funding acquisition (equal); investigation (equal); methodology (equal); project administration (equal); resources (equal); software (equal); supervision (equal); validation (equal); visualization (equal); writing – original draft (equal); writing – review and editing (equal). **Sylvia A Opanga:** Conceptualization (equal); data curation (equal); formal analysis (equal); funding acquisition (equal); investigation (equal); methodology (equal); project administration (equal); resources (equal); software (equal); supervision (equal); validation (equal); visualization (equal); writing – original draft (equal); writing – review and editing (equal). **David G Nyamu:** Conceptualization (equal); data curation (equal); formal analysis (equal); funding acquisition (equal); investigation (equal); methodology (equal); project administration (equal); resources (equal); software (equal); supervision (equal); validation (equal); visualization (equal); writing – original draft (equal); writing – review and editing (equal).

## FUNDING INFORMATION

There was no funding to conduct this study.

## CONFLICT OF INTEREST

The authors declare that they have no conflicts of interest.

## ETHICS STATEMENT

The study protocol was approved by the Kenyatta National Hospital/University of Nairobi Ethics and Research Committee (Approval No: P195/03/2021). After approval, permission to collect the data was obtained from the Health Information Department of KNH before the data collection from patients' medical records. The patients' names and addresses were not recorded during the data collection to ensure the patients' confidentiality.

## PATIENT CONSENT STATEMENT

Because the study was retrospective, we obtained participants' informed consent waivers from the Ethics committee.

## Data Availability

The datasets used and/or analyzed during the current study will be obtained from the corresponding author of this project.
